# The Effect of Changing Patterns of Obstetric Care in Scotland (1980–2004) on Rates of Preterm Birth and Its Neonatal Consequences: Perinatal Database Study

**DOI:** 10.1371/journal.pmed.1000153

**Published:** 2009-09-22

**Authors:** Jane E. Norman, Carole Morris, James Chalmers

**Affiliations:** 1 University of Edinburgh Centre for Reproductive Biology, Edinburgh, United Kingdom; 2 Information Services Division, NHS National Services Scotland, Edinburgh, United Kingdom; University of Oxford, United Kingdom

## Abstract

**Background:**

Rates of preterm birth are rising worldwide. Studies from the United States and Latin America suggest that much of this rise relates to increased rates of medically indicated preterm birth. In contrast, European and Australian data suggest that increases in spontaneous preterm labour also play a role. We aimed, in a population-based database of 5 million people, to determine the temporal trends and obstetric antecedents of singleton preterm birth and its associated neonatal mortality and morbidity for the period 1980–2004.

**Methods and Findings:**

There were 1.49 million births in Scotland over the study period, of which 5.8% were preterm. We found a percentage increase in crude rates of both spontaneous preterm birth per 1,000 singleton births (10.7%, *p*<0.01) and medically indicated preterm births (41.2%, *p*<0.01), which persisted when adjusted for maternal age at delivery. The greater proportion of spontaneous preterm births meant that the absolute increase in rates of preterm birth in each category were similar. Of specific maternal complications, essential and pregnancy-induced hypertension, pre-eclampsia, and placenta praevia played a decreasing role in preterm birth over the study period, with gestational and pre-existing diabetes playing an increasing role. There was a decline in stillbirth, neonatal, and extended perinatal mortality associated with preterm birth at all gestation over the study period but an increase in the rate of prolonged hospital stay for the neonate. Neonatal mortality improved in all subgroups, regardless of obstetric antecedent of preterm birth or gestational age. In the 28 wk and greater gestational groups we found a reduction in stillbirths and extended perinatal mortality for medically induced but not spontaneous preterm births (in the absence of maternal complications) although at the expense of a longer stay in neonatal intensive care. This improvement in stillbirth and neonatal mortality supports the decision making behind the 34% increase in elective/induced preterm birth in these women. Although improvements in neonatal outcomes overall are welcome, preterm birth still accounts for over 66% of singleton stillbirths, 65% of singleton neonatal deaths, and 67% of infants whose stay in the neonatal unit is “prolonged,” suggesting this condition remains a significant contributor to perinatal mortality and morbidity.

**Conclusions:**

In our population, increases in spontaneous and medically induced preterm births have made equal contributions to the rising rate of preterm birth. Despite improvements in related perinatal mortality, preterm birth remains a major obstetric and neonatal problem, and its frequency is increasing.

*Please see later in the article for the Editors' Summary*

## Abstract 2

Jane Norman and colleagues analyzed linked perinatal surveillance data in Scotland and find that between 1980 and 2004 increases in spontaneous and medically induced preterm births contributed equally to the rising rate of preterm births.

## Editors' Summary

**Background:**

Most pregnancies last about 40 weeks but increasing numbers of babies are being born preterm, before they reach 37 weeks of gestation (gestation is the period during which a baby develops in its mother). Nowadays in the US, for example, more than half a million babies arrive earlier than expected every year (1 in 8 babies). Although improvements in the care of newborn babies (neonatal care) mean that preterm babies are more likely to survive than in the past, preterm birth remains the single biggest cause of infant death in many developed countries, and many preterm babies who survive have long-term health problems and disabilities, particularly those born before 32 weeks of gestation. Preterm births can be spontaneous or medically induced. At present, it impossible to predict which mothers will spontaneously deliver early and there is no effective way to prevent these preterm births; medically induced early labor is undertaken when either the unborn baby or mother would be at risk if the pregnancy continued to full term.

**Why Was This Study Done?:**

Preterm birth rates need to be reduced, but before this can be done it is important to know how the causes of preterm birth, the numbers of preterm stillbirths, and the numbers of preterm babies who die at birth (neonatal deaths) or soon after (perinatal deaths) are changing with time. If, for example, the rise in preterm births is mainly due to an increase in medically induced labor and if this change in practice has reduced neonatal deaths, it would be unwise to try to reduce the preterm birth rate by discouraging medically induced preterm births. So far, data from the US and Latin America suggest that the increase in preterm births in these countries is solely due to increased rates of medically induced preterm births. However, in Europe and Australia, the rate of spontaneous preterm births also seems to be increasing. In this study, the researchers examine the trends over time and causes of preterm birth and of neonatal death and illness in Scotland over a 25-year period.

**What Did the Researchers Do and Find?:**

By searching a Scottish database of linked maternity records and infant health and death records, the researchers identified 1.49 million singleton births that occurred between 1980 and 2004 of which nearly 90,000 were preterm births. Over the study period, the rates of spontaneous and of medically induced preterm births per 1,000 births increased by 10.7% and 41.2%, respectively, but because there were more spontaneous preterm births than medically induced preterm births, the absolute increase in the rates of each type of birth was similar. Several maternal complications including preeclampsia (a condition that causes high blood pressure) and placenta previa (covering of the opening of the cervix by the placenta) played a decreasing role in preterm births over the study period, whereas gestational and preexisting diabetes played an increasing role. Finally, there was a decline in stillbirths and in neonatal and perinatal deaths among preterm babies, although more babies remained in the hospital longer than 7 days after birth. More specifically, after 28 weeks of gestation, stillbirths and perinatal deaths decreased among medically induced preterm births but not among spontaneous preterm births.

**What Do These Findings Mean?:**

These findings indicate that in Scotland between 1980 and 2004, increases in spontaneous and medically induced preterm births contributed equally to the rising rate of preterm births. Importantly, they also show that the increase in induced preterm births helped to reduce stillbirths and neonatal and perinatal deaths, a finding that supports the criteria that clinicians currently use to decide whether to induce an early birth. Nevertheless, preterm births still account for two-thirds of all stillbirths, neonatal deaths, and extended neonatal stays in hospital and thus cause considerable suffering and greatly increase the workload in neonatal units. The rates of such births consequently need to be reduced and, for Scotland at least, ways will have to be found to reduce the rates of both spontaneous and induced preterm births to achieve this goal while continuing to identify those sick babies who need to be delivered early to give them the best chance of survival.

**Additional Information:**

Please access these Web sites via the online version of this summary at http://dx.doi.org/10.1371/journal.pmed.1000153

- Tommys is a nonprofit organization that funds research and provides information on the causes and prevention of miscarriage, premature birth, and stillbirth
- The March of Dimes, a nonprofit organization for pregnancy and baby health, provides information on preterm birth (in English and Spanish)
- The Nemours Foundation, another nonprofit organization for child health, also provides information on premature babies (in English and Spanish)
- The US Centers for Disease Control and Prevention provides information on maternal and infant health (in English and Spanish)
- The US National Women's Health Information Center has detailed information about pregnancy, including a section on pregnancy complications
- MedlinePlus provides links to other information on premature babies and to information on pregnancy (in English and Spanish)

## Background

Preterm birth is the pre-eminent problem facing perinatologists in developed countries and has been defined as a major public health problem whose magnitude is increasing [1]. Although the secular trend is of an improvement in gestation specific outcomes (at least in terms of mortality) [2],[3], absolute rates of preterm birth are increasing in both the US [2] and in European countries such as the UK [4],[5] and Denmark [6]. Thus prematurity arising from preterm birth remains the biggest single cause of perinatal mortality and morbidity in most developed countries [7]. Governments and health care providers are increasingly concerned about this issue, and there have been calls for action from many bodies [1],[8].

Preterm birth may result from induced labour or operative delivery (medically induced preterm birth) in either the fetal or maternal interest (e.g., in the presence of intrauterine growth retardation or pre-eclampsia) or it may follow spontaneous preterm labour with or without preterm premature ruptured membranes [9]. If rates of preterm birth are to be reduced, a detailed understanding of both the temporal trends in the causes (obstetric antecedents) of preterm birth, and the neonatal morbidity and mortality associated with each of these causes is essential. Additionally, this information will determine the likely impact of treatment strategies (including novel therapies such as progesterone for the prevention of preterm birth), assist in service planning and in determining priorities for research and treatment, and last, but by no means least, help obstetricians, neonatologists, and the public in decision making around indicated preterm birth in high risk pregnancies. For example, an effective tocolytic or preterm preventive agent will help to reduce preterm birth occurring as a consequence of idiopathic preterm labour, but will not reduce elective/induced preterm delivery rates. Additionally, if the hypothesis that preterm birth rates are rising largely due to an increase in medically indicated preterm birth is correct, and that this increase in medically indicated preterm birth is associated with a decrease in perinatal mortality and morbidity, then extensive efforts to reduce elective preterm birth rates might be unwarranted or harmful.

Recent reports from the US have suggested that much of the increase in singleton preterm birth rates has resulted from rising rates of medically indicated preterm birth, and that spontaneous preterm birth rates are static or falling, especially amongst high risk ethnic groups [10],[11]. A fall in perinatal mortality in parallel with this rise in medically indicated preterm birth has been interpreted to mean that the rise in medically indicated preterm birth is appropriate and beneficial, and that this rise has (in part) caused the reduction in perinatal mortality [10]. In contrast, emerging data from Europe and Australia show that spontaneous preterm birth may also play a role [5],[6],[12].

The aim of this study was to determine the temporal trends in obstetric antecedents of preterm birth, and the temporal trends in neonatal mortality and morbidity associated with each of these antecedents during the period 1980 to 2004, using Scotland's comprehensive perinatal database [13]. We hypothesised that, in contrast to reports from the US and Latin America (which highlight an increase in elective delivery), increased rates of idiopathic spontaneous labour (with or without preterm premature membrane rupture [pPROM]) would be a major contributor to the increase in preterm birth rates observed in Scotland, even when adjusting for a change in maternal age over the study period. Additionally, we hypothesised that neonatal outcomes of preterm birth would show a progressive improvement over the time period studied. If our hypotheses are correct, then a major drive to reduce spontaneous preterm labour would be appropriate and would have the biggest impact in averting the adverse neonatal consequences of preterm birth.

## Methods

We explored the SMR02/SMR11/SBR/SSBID/GROS Birth Database (“The Linked Maternity and Neonatal Database”). This database contains linked maternity, neonatal, and stillbirth/infant death records, with records pertaining to mother and baby held together. The SMR02 (Scottish Morbidity Records 2) return is completed at the time of discharge of any patient from a Scottish maternity hospital and the level of completeness over the period studied is estimated to be in excess of 98%. There is also a facility for the data to be returned in the case of home births but this is thought to be less complete. However, home births in Scotland during this period comprised less than 1% of all births, so this is unlikely to be a source of significant error. SMR11 (now replaced by Scottish Birth Record, or SBR) are routine neonatal returns. SSBID is a record relating to Stillbirths and Infant Deaths, based on stillbirths and infant deaths that are registered with the General Register Office for Scotland (GROS). For each SSBID event, further information is sought from the relevant hospital. Because the data are based on registered events, it is unlikely that any cases are missed.

We examined records on singleton births between 1975 and 2004. Although we initially planned to report on data throughout this period, after an initial review we opted to confine our analysis to the period 1980–2004, excluding data from 1975–1979. The rationale for this was 2-fold: firstly, the coding changed from ICD8 to ICD9 in 1979 and many of the relevant codes do not map satisfactorily between these two systems, and secondly, the overall data quality in the early years of SMR02 appeared not to be adequate enough for this study, with problems such as augmentation with oxytocin probably being erroneously recorded as induction in a number of records.

We calculated the number and percentage of all singleton preterm births (live and stillbirths before 37 completed wk of gestation) and the number and percentage of singleton preterm births between 24 and 27 wk, 28 and 31 wk, and 32 and 36 wk. Gestational age at birth (as recorded on SMR02) is based on the clinician's best “guess” of estimated date of delivery, based largely on last menstrual period and ultrasound findings. The latter will have been used for confirmation in the majority of women—since the early 1990s, more than 95% of pregnant women in the UK (including Scotland) have had ultrasound in the first half of their pregnancy [14] (Professors Martin Whittle [Chair of the UK National Screening Committee] and Professor Andrew Calder [Professor of Obstetrics and Gynaecology, University of Edinburgh 1987–2009], personal communication). Any records missing the gestational age have been excluded from the analysis.

Thereafter, we calculated outcomes for 5 calendar year periods starting in 1980; i.e., from the period 1980–1984 up to 2000–2004. Initially preterm birth was classified according to its obstetric antecedents (see below for definition). These antecedents were used to subdivide preterm birth numbers and percentages. Outcomes were then further analysed by gestation at delivery (24–27 wk, 28–31 wk, 32–36 wk gestation).

The obstetric antecedents of preterm birth were defined as follows: spontaneous preterm labour with maternal complications, spontaneous preterm labour without maternal complications, pPROM with maternal complications, pPROM without maternal complications, indicated preterm delivery (labour induction or elective caesarean delivery) with maternal complications, and indicated preterm delivery without maternal complications. These antecedents are similar to those used by other investigators in this field [3]. We used the ICD codes for pPROM (658.1, 658.2 [ICD 9], O42 [ICD10]): with ICD 10 defining pPROM as preterm membrane rupture, which occurs more than 1 h prior to the onset of labour. Other ICD codes were used for maternal complications as follows: essential hypertension: 6420–6423 (ICD9) O10 (ICD 10); pregnancy induced hypertension: 6424, 6425, 6427, and 6429 (ICD 9) O11, O13, and O14 (ICD 10); eclampsia: 6246 (ICD 9),O15 (ICD 10); placenta praevia: 6410, 6411, 6635 (ICD 9), O44, O694 (ICD 10); abruption: 6412,6413,6418,6419 (ICD 9), O45,O441 (ICD 10); pre-existing diabetes: 250, 6480 (ICD 9) E10-14, O240-1, O243 (ICD 10); gestational diabetes: 6488 (ICD 9) O244, O249 (ICD 10). Any other maternal disease was classified as any other diagnosis code excluding delivery records containing those above and the following: 630–678, 760–779, all V codes (factors influencing health status and contact with health services [ICD 9]), O00–O99, all Z codes (factors influencing health status and contact with health services [ICD 10]).

We calculated secular trends in rates of preterm birth associated with these maternal complications. The relative contribution of each of the maternal complications to preterm birth was calculated for the 10-y period 1995–2004. Additionally we determined the following neonatal outcomes associated with preterm birth: birthweight, stillbirth, neonatal death, extended perinatal mortality (the rate of stillbirths and deaths within the first month of life per 1,000 live births), and the incidence of prolonged stay in the neonatal unit (defined as more than 7 d) [15]. The secular trend in incidence of these neonatal outcomes was calculated for all singleton preterm births, and then further subdivided by gestation at delivery (24–27 wk, 28–31 wk, 32–36 wk). Given the known change in 1992 in gestation used to define stillbirth, we analysed secular trends in stillbirth and extended perinatal mortality from 1995–1999 to 2000–2004, whereas secular trends in neonatal mortality were analysed over the whole of the study period, from 1980–1984 to 2000–2004. We looked at aggregate data from 1995–2004 to define the contribution of the obstetric antecedents of preterm birth to adverse neonatal outcomes, and calculated the contribution of the specific maternal complications.

Permission for record linkage and analysis of data for the purpose of this study was obtained from the Privacy Advisory Committee of NHS National Services Scotland.

### Statistical Analysis

Mantel-Haenszel chi-square (linear by linear association) test for trend was performed on temporal trends for the obstetric antecedents and subdivided by gestation groups to examine whether there was a linear relationship over the time period.

Univariate analysis was initially carried out to examine each individual confounding factor in relation to the outcomes of preterm birth, stillbirth, low birthweight, neonatal death, and prolonged stay in neonatal care. Thereafter, we used multivariate logistic regression modelling to examine the relationship between preterm birth, neonatal outcomes, and the six obstetric antecedents. All confounding factors including birth cohort period, deprivation, parity, smoking status (only recorded since 1993), health board of residence, age, and gestation at delivery (where appropriate) were entered into the model. The outcomes were considered as dichotomous variables and the covariates above categorised. The *p* values for all hypothesis tests were two-sided, and we set the significance at *p*<0.05. The goodness of fit of the logistic regression models was assessed using the Hosmer and Lemeshow test. We used SPSS 13.0 software package to conduct all the statistical analyses.

## Results

### Causes of Preterm Birth

There were 1,490,074 births over the study period, of which 86,723 (5.8%) were preterm (before 37 completed wk gestation). As previously shown, rates of preterm birth per 1,000 singleton births increased over the time period studied from 5.4% singleton live births in the 5-y period beginning 1980 to 6.3% in the 5-y period beginning 2000 [5]. This increase in preterm birth rates applied to each of the three gestation subgroups (24–27 wk, 28–31 wk, and 32–36 wk) although the greatest proportionate increase was in the 24–27 wk subgroup. When preterm birth was divided into spontaneous and medically induced, we noted a modest rise in the crude rates of spontaneous preterm birth per 1,000 singleton births (10.73%, *p*<0.01) but greater percentage increase in medically indicated preterm birth rates (41.47%, *p*<0.01, chi-squared test for linear trend) over the study period (Table 1). The increase in spontaneous preterm birth largely relates to an increase in preterm birth associated with pPROM (189.17%, *p*<0.01, chi-squared test for linear trend). Looking at gestational age subgroups, the rise in the rate of spontaneous preterm birth held for all gestation subgroups, whereas the relative increase in rates of elective/induced preterm delivery was greatest in the 24–27 wk gestation subgroup. There was a modest increase in rates of induced/elective preterm birth in the 32–36 wk gestation subgroup but a 22% decline in the 28–31 wk gestation subgroup. However, given the much larger contribution of the 32–36 wk gestation subgroup to overall numbers, there was a net increase of 34% in induced/elective deliveries in babies of gestational age 28–36 wk over the study period (Table 2).

**Table 1:** Number and rates of singleton preterm births in association with each of the major obstetric antecedents.

	Number					Rates per 1,000 Singleton Births						
Obstetric Antecedents	1980–1984	1985–1989	1990–1994	1995–1999	2000–2004	1980–1984	1985–1989	1990–1994	1995–1999	2000–2004	% Increase/Decrease	*p*(chi-square test for linear trend)
All singleton births	324,725	319,361	313,659	280,613	251,716							
All singleton preterm births	17,659	17,822	18,216	17,070	15,956	54.38	55.81	58.08	60.83	63.39	16.60%	<0.01
Spontaneous preterm birth	14,253	14,063	14,185	12,700	12,234	43.89	44.03	45.22	45.26	48.60	10.73%	<0.01
Spontaneous preterm births with pPROM	1,093	1,260	2,512	2,727	2,450	3.37	3.95	8.01	9.72	9.73	189.17%	<0.01
Spontaneous preterm births without pPROM	13,160	12,803	11,673	9,973	9,897	40.53	40.09	37.22	35.54	39.32	−2.98%	<0.01
Induction/elective caesarean delivery—preterm births	3,394	3,743	4,005	4,346	3,722	10.45	11.72	12.77	15.49	14.79	41.47%	<0.01

pPROM, premature rupture of membranes.

**Table 2:** Change in numbers and rates of preterm birth in association with each obstetric outcome with time.

		Number					Rate per 1000 Singleton Births						
Obstetric Outcome	Gestation (wk)	1980–1984	1985–1989	1990–1994	1995–1999	2000–2004	1980–1984	1985–1989	1990–1994	1995–1999	2000–2004	% Increase or Decrease	*p* (Chi-Square Test for Linear Trend)
**All singleton births**	All	324,725	319,361	313,659	280,613	251,716							
**Singleton preterm birth**	24–36	17,659	17,822	18,216	17,070	16,094	54.38	55.81	58.08	60.83	63.94	17.6%	<0.01
	24–27	597	654	988	947	973	1.84	2.05	3.15	3.37	3.87	110.3%	<0.01
	28–31	2,291	2,228	2,274	2,017	1,910	7.06	6.98	7.25	7.19	7.59	7.6%	<0.01
	32–36	14,771	14,940	14,954	14,106	13,211	45.49	46.78	47.68	50.27	52.48	0.15%	<0.01
**Spontaneous preterm births with pPROM**	24–36	1,093	1,260	2,512	2,727	2,450	3.37	3.95	8.01	9.72	9.73	189.2%	<0.01
	24–27	63	91	175	159	144	0.19	0.28	0.56	0.57	0.57	194.9%	<0.01
	28–31	196	221	375	342	308	0.60	0.69	1.20	1.22	1.22	102.7%	<0.01
	32–36	834	948	1,962	2,226	1,998	2.57	2.97	6.26	7.93	7.94	2.09%	<0.01
**Spontaneous preterm births without pPROM**	24–36	13,160	12,803	11,673	9,973	9,897	40.53	40.09	37.22	35.54	39.32	−3.0%	<0.01
	24–27	496	509	648	495	594	1.53	1.59	2.07	1.76	2.36	54.5%	<0.01
	28–31	1,589	1,466	1,442	1,248	1,295	4.89	4.59	4.60	4.45	5.14	5.1%	<0.01
	32–36	11,075	10,828	9,583	8,230	8,008	34.11	33.91	30.55	29.33	31.81	−0.07%	<0.01
**Induction/elective caesarean delivery**	24–36	3,394	3,743	4,005	4,346	3,722	10.45	11.72	12.77	15.49	14.79	41.5%	<0.01
	24–27	37	53	162	293	235	0.11	0.17	0.52	1.04	0.93	719.4%	<0.01
	28–31	505	538	452	427	305	1.56	1.68	1.44	1.52	1.21	−22.1%	<0.01
	32–36	2,852	3,152	3,391	3,626	3,182	8.78	9.87	10.81	12.92	12.64	0.44%	<0.01

pPROM, premature rupture of membranes.

**Figure 1:**
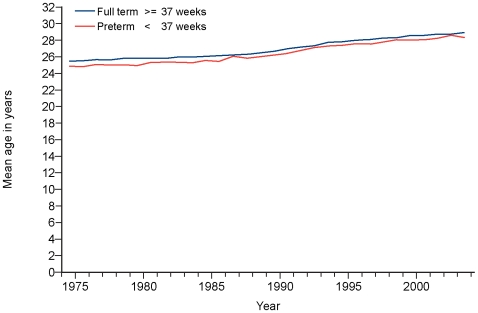
Change in mean maternal age at time of term or preterm birth, 1980–2004.

In view of the increase in maternal age of first pregnancy over the study period (Figure 1) [16],[17] and the known effects of maternal age on preterm birth rates, we calculated age standardised preterm birth rates and looked at their changes over time.

**Figure 2:**
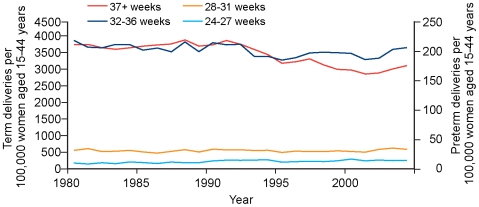
Spontaneous births per 100,000 women of reproductive age, 1980–2004.

**Figure 3:**
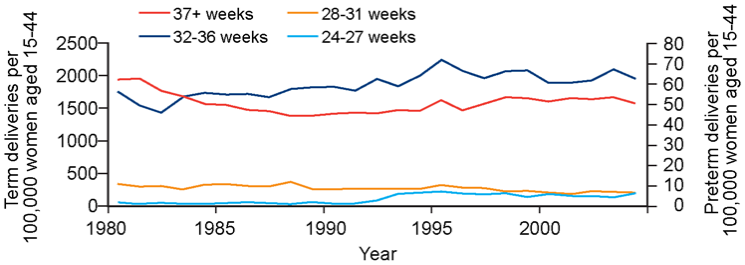
Induced/elective births per 100,000 women of reproductive age, 1980–2004.

Maternal age standardised rates of spontaneous deliveries (preterm and term) and induced deliveries (preterm and term) for the period 1980 to 2004 are shown in Figures 2 and 3. There was a small (2.3%) decline in maternal age adjusted spontaneous preterm birth rate on a background of a greater decline (17.1%) in maternal age adjusted spontaneous birth at term (Figure 2). There was a modest (10.5%) increase in maternal age adjusted induced/elective preterm birth rate, which contrasted with a 23.4% reduction in age adjusted induced/elective term birth rate (Figure 3).

Maternal complications were present in 24.3% of preterm births. Essential and pregnancy induced hypertension, pre-eclampsia, and placenta praevia played a decreasing role in preterm birth over the study period (*p*<0.01, chi-squared test for linear trend). In contrast, there was over a 7-fold increase in preterm birth associated with pre-existing diabetes, and a 4-fold increase in preterm birth associated with gestational diabetes (*p*<0.01) (Table 3).

**Table 3:** Change in maternal complications associated with preterm births 1980 to 2004.

	Number					Rate per 1000 singleton births						
Maternal Complication	1980–1984	1985–1989	1990–1994	1995–1999	2000–2004	1980–1984	1985–1989	1990–1994	1995–1999	2000–2004	% increase/decrease	P (chi-square test for linear trend)
All singleton preterm births^a^	17,659	17,822	18,216	17,070	15,956							
Essential hypertension	150	172	127	130	114	0.85	0.97	0.70	0.76	0.71	−15.89%	**<0.01**
Pregnancy induced hypertension	2,554	2,773	3,090	2,428	1,836	14.46	15.56	16.96	14.22	11.51	−20.44%	**<0.01**
Eclampsia	61	43	57	56	29	0.35	0.24	0.31	0.33	0.18	−47.38%	**<0.01**
Placenta praevia	610	649	653	577	288	3.45	3.64	3.58	3.38	1.80	−47.75%	**<0.01**
Pre-existing diabetes	38	11	134	286	286	0.22	0.06	0.74	1.68	1.79	732.96%	**<0.01**
Gestational diabetes	29	47	80	119	138	0.16	0.26	0.44	0.70	0.86	426.65%	**<0.01**
Other conditions^b^	0	0	0	6	0	0.00	0.00	0.00	0.04	0.00		

a Either with or without maternal complications.

b Other conditions exclude all delivery records with an occurrence of any of the above conditions.

Given the smaller numbers of preterm births associated with maternal complications, data from the 10-y period (1995–2004) were aggregated to examine the obstetric conditions relevant to preterm birth within each gestational cohort. Overall, pregnancy induced hypertension was the commonest obstetric antecedent of preterm births, being present in nearly 13% of preterm births. The second commonest obstetric antecedent was abruption, being present in 9% of preterm births. However, there was a clear difference in the leading obstetric antecedent at each gestation: abruption was the commonest obstetric antecedent of preterm birth in the shortest gestation cohort (associated with 17.7% of preterm births at 24–27 wk gestation), with pregnancy induced hypertension being the commonest obstetric antecedent of preterm birth at the longer two gestation periods (16.7% at 28–31 wk gestation, and 12.5% at 32–36 wk gestation). Abruption and/or pregnancy induced hypertension combined were associated with 25.1% of singleton preterm births, compared with 12.6% of all singleton births.

The adjusted odds of preterm birth in association with specific maternal complications (both unadjusted and after adjusting for deprivation, parity, smoking, health board of residence, and maternal age and birth cohort period), in comparison with having no maternal complications, are shown in Table 4. The odds of preterm birth are increased in association with all maternal disease, and were greatest in association with eclampsia (adjusted OR 8.94 [95% CI 6.93–11.54]), placenta praevia (adjusted OR 8.42 [95% CI 7.81–9.08]), and pre-existing diabetes (adjusted OR 6.67 [95% CI 6.03–7.37]). These maternal antecedents increased the risk of both spontaneous preterm labour and induced/elective preterm birth.

**Table 4:** Logistic regression modelling for the outcome preterm birth (<37 wk).

	Unadjusted					Adjusted				
Group	*N*	OR	Lower 95% CI	Upper 95% CI	*p*-Value	*N*	OR	Lower 95% CI	Upper 95% CI	*p*-Value
**All women**										
Essential hypertension	7,188	1.73	1.60	1.88	<0.01	1,890	2.72	2.38	3.10	<0.01
Pregnancy induced hypertension	138,011	1.74	1.70	1.77	<0.01	42,960	2.52	2.44	2.60	<0.01
Eclampsia	723	8.42	7.22	9.82	<0.01	259	8.94	6.93	11.54	<0.01
Placenta praevia	9,374	7.02	6.71	7.34	<0.01	3,210	8.42	7.81	9.08	<0.01
Abruption	51,836	4.69	4.58	4.79	<0.01	18,352	4.78	4.61	4.97	<0.01
Pre-existing diabetes	2,551	6.83	6.27	7.44	<0.01	1,893	6.67	6.03	7.37	<0.01
Gestational diabetes	5,569	1.29	1.17	1.43	<0.01	2,832	1.66	1.46	1.89	<0.01
Other non-obstetric conditions	137	0.74	0.33	1.67	0.46	107	0.62	0.23	1.68	0.34
**Spontaneous labour only**										
Essential hypertension	3,086	1.88	1.68	2.10	<0.01	716	3.26	2.69	3.95	<0.01
Pregnancy induced hypertension	61,365	1.83	1.78	1.88	<0.01	19,064	2.78	2.66	2.90	<0.01
Eclampsia	424	10.38	8.56	12.59	<0.01	161	10.39	7.58	14.24	<0.01
Placenta praevia	4,753	11.57	10.92	12.26	<0.01	1,648	16.08	14.56	17.75	<0.01
Abruption	34,664	5.40	5.26	5.54	<0.01	12,400	5.48	5.25	5.72	<0.01
Pre-existing diabetes	843	16.26	14.20	18.62	<0.01	618	16.04	13.66	18.83	<0.01
Gestational diabetes	2,754	1.66	1.47	1.88	<0.01	1,304	2.47	2.11	2.90	<0.01
Other non-obstetric conditions	94	0.64	0.23	1.73	0.38	71	0.41	0.10	1.69	0.22
**Induced labour/elective caesarean only**										
Essential hypertension	4,095	2.04	1.82	2.28	<0.01	1,169	2.89	2.41	3.47	0.16
Pregnancy induced hypertension	76,547	2.31	2.24	2.39	<0.01	23,847	3.11	2.95	3.28	<0.01
Eclampsia	296	6.57	5.01	8.63	<0.01	97	6.94	4.36	11.04	<0.01
Placenta praevia	4,612	4.11	3.79	4.46	<0.01	1,557	3.66	3.17	4.23	<0.01
Abruption	17,139	3.26	3.11	3.42	<0.01	5,934	3.18	2.93	3.45	<0.01
Pre-existing diabetes	1,698	4.97	4.39	5.63	<0.01	1267	4.65	4.01	5.39	<0.01
Gestational diabetes	2,803	1.05	0.88	1.26	0.56	1,521	1.14	0.90	1.44	0.28
Other non-obstetric conditions	43	1.09	0.26	4.52	0.90	36	1.18	0.28	4.94	0.82

Unadjusted and adjusted ORs with 95% CI and *p*-value in relation to maternal conditions. Adjusted ORs have been adjusted for the following factors: deprivation, parity smoking, health board of residence, and maternal age. *N*, Number of preterm births to a mother with the specific condition included in the analysis. (Note: referent category for each condition is not having the specific condition.)

### Neonatal Outcomes

#### Stillbirth and neonatal death

The OR for stillbirth, both unadjusted and after adjustment for parity, smoking, deprivation, birth cohort period, and health board of residence is shown in Table 5. Over 90% of these stillbirths were antepartum events. Unadjusted ORs for stillbirth were greater in babies born at earlier gestations (OR of 15.71 at 32–36 wk, 77.15 at 28–31 wk, and 101.88 at 24–28 wk compared with term babies [95% CI 15.00–16.46, 72.94–81.59, and 94.28–110.09, respectively]), to women with low or high parity (ORs least in those who were para 1: 0.69 [95% CI 0.66–0.72] compared with nulliparous women), to women who smoked (OR 1.54 [95% CI 1.45–1.64] compared with non-smokers), and to women from a higher deprivation quintile (OR 1.62 [95% CI 1.51–1.72] in the most deprived compared with the least deprived quintile). The majority of stillbirths occurred in association with spontaneous preterm labour in the absence of maternal complications and without pPROM; although the OR of stillbirth was greatest in association with induced/elective preterm delivery.

**Table 5:** Logistic regression modelling for neonatal outcomes.

	Unadjusted					Adjusted				
Outcome	*N*	OR	95% CI		*p*-Value	*N*	OR	95% CI		*p*-Value
			Lower	Upper				Lower	Upper	
**Low birth weight (<2,500 g)**										
Spontaneous preterm birth^a^ without maternal complications	41,816	1.00				19,217	1.00			
Spontaneous preterm birth^a^ with maternal complications	15,690	2.13	2.05	2.22	<0.05	7,079	2.02	1.89	2.15	<0.05
Preterm birth with pPROM without maternal complications	10,388	1.31	1.25	1.36	<0.05	6,169	1.14	1.07	1.22	<0.05
Preterm birth with pPROM with maternal complications	1,723	1.87	1.69	2.08	<0.05	951	1.31	1.12	1.52	<0.05
Preterm induction/elective caesarean delivery without maternal complications	8,646	1.51	1.44	1.58	<0.05	5,070	1.46	1.36	1.56	<0.05
Preterm induction/elective caesarean delivery with maternal complications	8,500	1.69	1.61	1.78	<0.05	3,902	1.72	1.60	1.86	<0.05
**Stillbirth (cohort includes 1995–2004 births only)**										
Spontaneous preterm birth^a^ without maternal complications	14,797	1.00				13,227	1.00			
Spontaneous preterm birth^a^ with maternal complications	5,073	1.50	1.24	1.82	<0.05	4,294	1.01	0.81	1.27	0.93
Preterm birth with pPROM without maternal complications	5,382	0.42	0.31	0.57	<0.05	4,606	0.42	0.30	0.59	<0.05
Preterm birth with pPROM with maternal complications	747	1.08	0.66	1.77	0.76	614	0.50	0.27	0.91	<0.05
Preterm induction/elective caesarean delivery without maternal complications	4,367	13.23	11.58	15.11	<0.05	3,987	19.84	16.77	23.47	<0.05
Preterm induction/elective caesarean delivery with maternal complications	2,752	5.96	5.07	7.01	<0.05	2,462	9.86	8.05	12.07	<0.05
**Neonatal death**										
Spontaneous preterm birth^a^ without maternal complications	41,816	1.00				19,217	1.00			
Spontaneous preterm birth^a^ with maternal complications	15,690	1.17	1.07	1.27	<0.05	7,079	0.88	0.74	1.04	0.13
Preterm birth with pPROM without maternal complications	10,388	0.89	0.80	1.00	<0.05	6,169	0.88	0.72	1.09	0.24
Preterm birth with pPROM with maternal complications	1,723	1.33	1.08	1.65	<0.05	951	1.02	0.71	1.46	0.92
Preterm induction/elective caesarean delivery without maternal complications	8,646	0.60	0.52	0.69	<0.05	5,070	0.38	0.29	0.50	<0.05
Preterm induction/elective caesarean delivery with maternal complications	8,500	0.52	0.45	0.60	<0.05	3,902	0.32	0.23	0.46	<0.05
**Prolonged stay in neonatal care (>7 days)**										
Spontaneous preterm birth^a^ without maternal complications	34,144	1.00				12,411	1.00			
Spontaneous preterm birth^a^ with maternal complications	13,465	1.99	1.91	2.07	<0.05	5,251	2.16	2.02	2.32	<0.05
Preterm birth with pPROM without maternal complications	7,826	1.27	1.20	1.33	<0.05	4,092	1.17	1.08	1.26	<0.05
Preterm birth with pPROM with maternal complications	1,402	1.95	1.75	2.17	<0.05	712	1.72	1.47	2.02	<0.05
Preterm induction/elective caesarean delivery without maternal complications	6,419	0.79	0.74	0.84	<0.05	3,049	0.68	0.62	0.75	<0.05
Preterm induction/elective caesarean delivery with maternal complications	7,436	1.43	1.36	1.51	<0.05	2,980	1.23	1.13	1.35	<0.05

Unadjusted and adjusted ORs with 95% CI and *p* values in relation to maternal conditions.

a In the absence of pPROM.

Unadjusted ORs for neonatal death were also greater in babies born at earlier gestations, to nulliparous women, to smokers, and to women of a high deprivation quintile. In contrast to the association between elective preterm delivery and stillbirth, the risk of neonatal death was lower in babies born preterm following elective/induced delivery, compared to those born after spontaneous preterm labour (Table 5).

Looking at secular trends over the study period, there was only a modest reduction in preterm birth associated stillbirth (prevalence ratio 0.9 in 2000–2004 compared with 1995–1999) but a greater decline in preterm birth associated neonatal death (prevalence ratio 0.43 in 2000–2004 compared with 1980–1984) (Table 6). Looking at obstetric causes of preterm birth, in the absence of maternal complications, we found a reduction in stillbirth and extended perinatal mortality for medically induced but not spontaneous preterm births at gestations of 28 wk and above although at the expense of a longer stay in neonatal intensive care.

**Table 6:** Temporal trends in neonatal outcomes (unadjusted prevalence ratio).

Outcome	Gestation Period (wk)			
	24–36	24–27	28–31	32–36
**Stillbirths** (relative prevalence, 2000–2004 compared with 1995–1999)				
**All singleton preterm births**	**0.90**	**0.98**	**0.81**	**0.90**
Spontaneous preterm birth^a^ without maternal complications	1.25	1.10	1.21	1.47
Spontaneous preterm birth^a^ with maternal complications	0.84	0.78	0.84	0.89
Preterm premature membrane rupture without maternal complications	0.80	0.53	1.30	1.49
Preterm premature membrane rupture with maternal complications	0.46	0.56	0.00	0.00
Induction/elective caesarean delivery without maternal complications	1.01	1.30	0.84	0.93
Induction/elective caesarean delivery with maternal complications	0.50	0.46	0.47	0.54
Induction/elective caesarean delivery (overall)	0.86	1.04	0.73	0.81
**Neonatal deaths** (relative prevalence, 2000–2004 compared with 1980–1984)				
**All singleton preterm births**	**0.43**	**0.67**	**0.27**	**0.35**
Spontaneous preterm birth^a^ without maternal complications	0.28	0.40	0.19	0.20
Spontaneous preterm birth^a^ with maternal complications	0.29	0.62	0.11	0.23
Preterm premature membrane rupture without maternal complications	0.69	1.39	0.34	0.23
Preterm premature membrane rupture with maternal complications	1.14	1.47	0.86	0.97
Induction/elective caesarean delivery without maternal complications	0.45	0.64	0.23	0.53
Induction/elective caesarean delivery with maternal complications	0.07	0.09	0.00	0.15
Induction/elective caesarean delivery (overall)	0.20	0.25	0.06	0.33
**Stillbirths and neonatal deaths (extended perinatal)** (relative prevalence, 2000–2004 compared with 1995–1999)				
**All singleton preterm births**	**0.90**	**0.96**	**0.84**	**0.89**
Spontaneous preterm birth^a^ without maternal complications	1.03	1.02	1.03	1.05
Spontaneous preterm birth^a^ with maternal complications	0.73	0.68	0.64	0.87
Preterm premature membrane rupture without maternal complications	0.65	0.70	0.73	0.41
Preterm premature membrane rupture with maternal complications	0.83	0.70	0.00	0.84
Induction/elective caesarean delivery without maternal complications	0.98	1.25	0.82	0.90
Induction/elective caesarean delivery with maternal complications	0.49	0.43	0.45	0.57
Induction/elective caesarean delivery (overall)	0.84	0.99	0.72	0.81
**Prolonged stay in neonatal care** (relative prevalence, 1995–1999 to 1980–1984)				
**All singleton preterm births**	**1.57**	**3.79**	**2.31**	**1.38**
Spontaneous preterm birth^a^ without maternal complications	0.85	1.64	1.25	0.76
Spontaneous preterm birth^a^ with maternal complications	1.22	2.89	1.74	1.03
Preterm premature membrane rupture without maternal complications	2.31	5.79	3.03	1.99
Preterm premature membrane rupture with maternal complications	1.74	4.17	2.76	1.31
Induction/elective caesarean delivery without maternal complications	1.41	3.86	2.08	1.34
Induction/elective caesarean delivery with maternal complications	0.82	1.93	0.85	0.80
Induction/elective caesarean delivery (overall)	1.02	2.57	1.09	1.00

a In the absence of pPROM.

#### Prolonged stay in hospital

Smoking, deprivation, and extremes of parity had similar adverse effects on prolonged stay in hospital as they did on stillbirth (unpublished data). Adjusted OR showed that, after adjustment for deprivation, parity, smoking, birth cohort period, and health board of residence, and in the absence of maternal complications, prolonged stay was less common in babies born preterm following elective/induced delivery compared with babies born preterm following spontaneous preterm labour (adjusted OR 0.68 [95% CI 0.62–0.75]) (Table 5).

Looking at secular trends, there was an increase in the incidence of prolonged stay in hospital in the period 1995–1999 compared with 1980–1984 (Table 6). This is consistently true for preterm births up to 32 wk gestation following all obstetric antecedents (with the minor exception of induced/elective preterm delivery with maternal complications).

#### Growth restriction

Babies who were small for gestational age (SGA) (*z* score<2) were more common amongst preterm compared with term births (proportions 2.18% and 1.82%, respectively) (unadjusted OR 1.11 [95% CI 1.06–1.16], *p*<0.01). The adverse effects of deprivation, smoking, and nulliparity on the incidence of SGA were similar to their effects on stillbirth and prolonged hospital stay. After adjustment for parity, deprivation, smoking, birth cohort period, and health board of residence, preterm birth was paradoxically associated with a reduction in odds of SGA. Looking at obstetric antecedents, in the absence of maternal complications, babies born as a result of induced/elective preterm birth were at an increased risk of being SGA after adjustment whereas those born preterm following pPROM were at reduced risk (Table 7).

**Table 7:** Logistic regression modelling for outcome of SGA (*z* score<2).

	Unadjusted					Adjusted				
	Number	OR	95% CI		Sig.	Number	OR	95% CI		Sig.
Obstetric Antecedents			Lower	Upper				Lower	Upper	
Spontaneous preterm birth^a^ without maternal complications	41,816	1.00				16,554	1.00			
Spontaneous preterm birth^a^ with maternal complications	15,690	1.36	1.16	1.59	<0.05	6,077	1.19	0.88	1.62	0.26
Preterm birth with pPROM without maternal complications	10,388	0.29	0.20	0.41	<0.05	5,468	0.19	0.10	0.37	<0.05
Preterm birth with pPROM with maternal complications	1,723	0.32	0.14	0.71	<0.05	830	0.42	0.13	1.33	0.14
Preterm induction/elective caesarean delivery without maternal complications	8,646	4.20	3.66	4.83	<0.05	4,593	3.98	3.12	5.06	<0.05
Preterm induction/elective caesarean delivery with maternal complications	8,500	2.21	1.87	2.61	<0.05	3,339	2.07	1.50	2.85	<0.05

Unadjusted and adjusted ORs with 95% CI and *p* values in relation to maternal conditions. ORs have been adjusted for deprivation, parity, smoking, health board of residence, and cohort birth period. Cohort includes preterm births <37 wk gestation only, 1980 to 2004 inclusive.

a In the absence of pPROM.

#### Contribution of categories of obstetric antecedents of preterm birth to adverse neonatal outcomes

The contribution of the categories of each of the obstetric antecedents of preterm birth to adverse neonatal outcomes, together with their overall contribution in terms of percentages of preterm birth, is shown in Table 8.

**Table 8:** Contribution of preterm birth and subtypes to neonatal events, data from 1995–2004.

Group	Number of Births	Births as a Percentage of All Births	Number of Stillbirths	Stillbirths as a Percentage of All Stillbirths	Number of Neonatal Deaths	NND as a Percentage of All NND	Number of Babies with Prolonged Stay in Hospital^a^	Prolonged Stay in Hospital as Percentage of All Prolonged Stays in Hospital^a^
All singleton births	534,386		2,720		1,241		7,167	
All singleton preterm births	33,333	6.2	1,818	66.8	805	64.9	4,832	67.4
Overall spontaneous PTB	25,205	4.7	512	18.8	708	57.1	3,835	53.5
Overall pPROM PTB	6,156	1.2	65	2.4	162	13.1	903	12.6
Overall induced elective PTB	8,079	1.5	1,304	47.9	95	7.7	987	13.8
Overall maternal complications			489	18.0	212	17.1	1,863	26.0

PTB, preterm birth; NND, neonatal death.

a Data on prolonged stay in hospital for 1995–1999 only.

## Discussion

At the outset of this study, we hypothesised that, in contrast to reports from the US and Latin America (which highlight an increase in elective delivery), increased rates of idiopathic spontaneous labour (with or without pPROM) would be a major contributor to the increase in preterm birth rates observed in Scotland. Our hypotheses were partially correct—although the percentage increase in preterm birth rates was greatest in the elective/induced category (a 41% increase over the study period), rates of preterm birth were also rising in the spontaneous preterm birth category (a 10% rise over the study period). These changes persist when adjusted for maternal age: a progressive rise in the proportion of preterm births (in both the elective and spontaneous category) would have occurred even if there had been no change in maternal age over the study period. Although the percentage rise in elective/induced preterm births is greater than that in spontaneous preterm births (with and without pPROM), the absolute increase in the rate of preterm births is similar in each group (4.24 compared with 4.71 per 1,000 singleton births, respectively [Table 1]). Thus, in our population, increases in spontaneous and elective/induced preterm births are making equal contributions to the rise in the rate of preterm births. Our results showing an increase in both elective/induced and spontaneous preterm birth rates contrast with those of Ananth and colleagues, who showed a 50% increase in the rate of medically indicated preterm birth in the US from 1989 to 2001, but a 5%–25% decline in spontaneous preterm birth (with and without pPROM) [10], and those of Barros and Velez Mdel, who showed a 80% increase in medically indicated preterm birth in Latin America between 1985 and 2000 due to elective induction/delivery but again a decline in spontaneous preterm birth and that associated with pPROM [3]. The discrepancy with the Barros paper may in part relate to the fact that we included women with gestational or pre-existing diabetes in contrast to Barros and Velez Mdel, who excluded them, especially since the contribution of both of these complications to preterm birth (in our population) increased significantly over the study period. Our results are in keeping with those of Langoff Roos et al. [6] and Tracy et al. [12], who showed a rise in spontaneous preterm deliveries over a 10-y period in both populations as a whole, and in specially constructed “standard” populations of low risk women.

The findings that pregnancy induced hypertension and abruption are the commonest maternal complications preceding preterm birth are not unexpected. However, the decline in rates of preterm birth in association with essential hypertension, pregnancy induced hypertension, eclampsia, and placenta praevia over the study period was not anticipated. It could imply either better obstetric management of these conditions (in that their severity is reduced, and the need for elective preterm delivery or the triggering of spontaneous preterm labour is lower), it could imply a greater willingness of obstetricians to manage these conditions conservatively, or it could be an artefact of varying completeness of coding. In contrast, we observed a 4- to 7-fold increase in preterm deliveries associated with pre-existing and gestational diabetes. The reasons for the increased contribution of diabetes to preterm delivery are likely to be multifactorial: greater numbers of women with pre-existing (both Type I and Type II) diabetes may be getting pregnant, ascertainment of gestational diabetes may be getting better, and the true incidence of gestational diabetes may be rising as obesity rates rise in pregnancy. The fact that gestational diabetes increased the odds of spontaneous preterm labour was somewhat of a surprise, and has not (to our knowledge) been highlighted in previous studies. Whatever the reason for the association between pre-existing and gestational diabetes and preterm birth, it suggests that treatment and management of diabetes during pregnancy will be a key issue in inhibiting any further rise in preterm birth rates, particularly given that the maternal condition associated with the greatest odds of preterm birth is pre-existing diabetes.

The reason for the increase in rates of induced/elective preterm birth in the absence of maternal complications is unclear. Induced/elective preterm delivery in this scenario implies detection of a fetal complication. However, there have been no new methods of fetal surveillance over this time period: indeed the current recommended tools for surveillance of the small-for-dates fetus are umbilical Doppler ultrasound and fetal biometry—both of which tools have been available for the majority of the study period [18]. There has been no change in prevalence of small-for-dates fetuses in the Scottish population over the study period [19], so it appears that obstetricians have had a lower threshold to act on information from surveillance strategies and to electively deliver the baby. This increased enthusiasm for induced/elective preterm may be in part due to the known improvement in neonatal mortality in all gestation subgroups over the study period.

In terms of spontaneous preterm labour in the absence of maternal complications, again there has been no systematic change in practice over the time period of the study that is likely to contribute to the increase in rates. Current UK guidelines suggest that it is reasonable not to use tocolytic agents given their lack of effect on outcomes or preterm delivery rates overall [20], but given their inefficacy, it is unlikely that any change in tocolytic use will have affected spontaneous preterm delivery rates.

A caveat to any study of this type has to be that we are relying on routinely collected data for our analysis, with issues of data quality and of other confounders that we were unable to measure. Although we did not formally examine SMR2 data quality for the purposes of this project, previous studies have confirmed that it is adequate for analyses of the type reported here. For example, an evaluation over a 6-mo period in 1996–1997 compared a 5% sample of paper records with the database. This showed that most fields had less than 2% errors. However, there was a 5.6% error rate in the estimated gestation, 6.4% error in induction of labour, 13.5% error in duration of labour, and a 10%–20% error rate in the recording of the main ICD diagnostic codes [21]. For these minor errors to have contributed to the secular trends seen, they would have to have caused systematic bias, which seems unlikely. A potential confounder that has been raised in many population studies of preterm birth rates is the increasing use of early ultrasound to more accurately date gestational age. We do not believe that this will have had an effect here: the trends observed have been consistent over time, and there will have been very little change in the proportion of pregnancies dated using first trimester ultrasound over the last 15-y period of this study. Another potential confounder that we were unable to address is ethnic origin: this is potentially important given the increased rates of preterm birth in African American compared with white American women. Again, we do not believe this to be relevant to our study: in the 2001 census nearly 98% of the population of Scotland was white, so it is unlikely that any potential increases in the small proportion of nonwhite inhabitants in the period 1980–2004 will have impacted on preterm delivery rates.

The results of our study show that to reverse the rising trend of preterm birth in Scotland, policies and research strategies should be focussed on both spontaneous and elective/induced preterm deliveries: different approaches will be required, but both are important. They also emphasise an emerging theme in the epidemiology of preterm birth in that although preterm birth rates are rising in all developed countries, the rise appears to be driven largely by rises in elective/induced preterm birth in the US and South America, but by an increase in both elective/induced and spontaneous preterm births in Europe and Australia.

The data supported our second hypothesis: that neonatal outcomes of preterm birth would show a progressive improvement over the time period studied. There was a 55% reduction in risk of neonatal death and a 10% reduction in risk of both stillbirth and extended perinatal death over the study period. However, these improved outcomes come at a cost to the health service with a 57% increase in the risk of prolonged stay in hospital for the neonate over the study period. These data are in keeping with those from other groups, showing improved perinatal outcomes in association with preterm birth over the time period of our study. The improvement in stillbirth neonatal mortality in the elective/induced preterm birth group at gestations of 28 wk and greater does support the decision making behind the rising trend of elective/induced preterm birth. It implies that sick babies are being appropriately identified, and rather than being stillborn or dying shortly after birth as they were in earlier time cohorts, they are delivered and survive. Whatever the specific indication for the elective/induced preterm delivery, the fact that elective/induced preterm babies are more likely than those delivered spontaneously to be SGA suggests that their in utero environment is compromised and has restricted their growth. However, once delivered alive, somewhat surprisingly these babies do better than those delivered spontaneously (in the absence of maternal complications in both groups) in terms of lower neonatal mortality and a lower incidence of prolonged hospital stay.

Our data showing lower neonatal mortality in electively delivered babies are somewhat different to those of Barros and Velez Mdel, who showed that babies born following medically indicated preterm birth, even in the absence of maternal complications, had higher neonatal mortality than those delivered following spontaneous preterm labour [3], and to those of Villar and colleagues, who showed that babies delivered electively preterm were more likely to have a prolonged stay in the neonatal unit [15]. This difference is even more surprising given that our population included women with diabetes whereas Barros and Villar excluded them. In contrast our data showing that the presence of maternal complications significantly increased the risk of neonatal mortality is in agreement with others in the literature [3],[15].

The rationale for poorer neonatal outcomes following spontaneous rather than elective preterm delivery may be that intrauterine infection, which often triggers spontaneous preterm labour, continues to have an adverse effect on the neonate after delivery. This likely lower neonatal mortality in electively delivered babies compared with those delivering spontaneously should be taken into account by obstetricians and paediatricians making decisions about elective preterm delivery of individual babies (especially those where delivery is indicated in the fetal interest and where there is no maternal compromise).

To summarise the neonatal outcomes of this study, the secular trend is of a reduction in mortality associated with preterm birth, supporting the decision making behind elective/induced preterm delivery, particularly at gestations of 28 wk and above. Although babies born electively (and alive) tend to be smaller than those delivering spontaneously, their outcomes tend to be at least as good. In contrast, the rising tide of antepartum stillbirth, which increasingly appears to be a factor in triggering elective preterm birth (in the absence of maternal complications), has been noted elsewhere, suggesting that research into this issue is needed. Lastly, a common theme throughout our data was that smoking and social deprivation both continue to play a significant role in the aetiology of preterm birth and are major risk factors for adverse neonatal outcomes.

## Abbreviations

GROS: General Register Office for Scotland
pPROM: preterm premature membrane rupture
SBR: Scottish Birth Record
SGA: small for gestational age
SMR02: Scottish Morbidity Records 2

## References

[1] Institute of Medicine of the National Academies (2007). Preterm birth: causes consequences and prevention.

[2] Martin JA, Hamilton BE, Sutton PD, Ventura SJ, Menacker F (2006). Births: final data for 2004.. Natl Vital Stat Rep.

[3] Barros FC, Velez Mdel P (2006). Temporal trends of preterm birth subtypes and neonatal outcomes.. Obstet Gynecol.

[4] Department of Health (2008). NHS Maternity Statistics England 2002–2003.

[5] Chalmers J (2006). Preterm deliveries in Scotland.. http://www.bmj.com/cgi/eletters/322/7547/937#132366.

[6] Langhoff-Roos J, Kesmodel U, Jacobsson B, Rasmussen S, Vogel I (2006). Spontaneous preterm delivery in primiparous women at low risk in Denmark: population based study.. BMJ.

[7] Confidential enquiry on maternal and child health (2007). Perinatal mortality 2005..

[8] Preterm birth: crisis and opportunity (2006). Lancet.

[9] Goldenberg RL, Culhane JF, Iams JD, Romero R (2008). Epidemiology and causes of preterm birth.. Lancet.

[10] Ananth CV, Joseph KS, Oyelese Y, Demissie K, Vintzileos AM (2005). Trends in preterm birth and perinatal mortality among singletons: United States, 1989 through 2000.. Obstet Gynecol.

[11] Demissie K, Rhoads GG, Ananth CV, Alexander GR, Kramer MS (2001). Trends in preterm birth and neonatal mortality among blacks and whites in the United States from 1989 to 1997.. Am J Epidemiol.

[12] Tracy SK, Tracy MB, Dean J, Laws P, Sullivan E (2007). Spontaneous preterm birth of liveborn infants in women at low risk in Australia over 10 years: a population-based study.. BJOG.

[13] Scottish Programme for Clinical Effectiveness in Reproductive Health (2007). Scottish perinatal and infant mortality and morbidity report (SPIMMR) 2006.

[14] Campbell S, Soothill P, Chervenak F, Isaacson G, Campbell S (1993). Detection and management of intrauterine growth retardation.. Ultrasound in obstetris and gynaecology.

[15] Villar J, Abalos E, Carroli G, Giordano D, Wojdyla D (2004). Heterogeneity of perinatal outcomes in the preterm delivery syndrome.. Obstet Gynecol.

[16] Office of National Statistics (2004). Birth statistics England and Wales 2002..

[17] Information Services Division NHS Scotland (2007). Births and babies..

[18] RCOG Guidelines and Audit Group (2002). The investigation and management of the small-for-dates fetus.

[19] Bonellie S, Chalmers J, Gray R, Greer I, Jarvis S (2008). Centile charts for birthweight for gestational age for Scottish singleton births.. BMC Pregnancy and Childbirth.

[20] Royal College of Obstericians and Gynaecologists (2002). Tocolytic drugs for women in preterm labour.

[21] Smith GC, Pell JP, Dobbie R (2003). Caesarean section and risk of unexplained stillbirth in subsequent pregnancy.. Lancet.

